# Alterations in the neural circuits from peripheral afferents to the spinal cord: possible implications for diabetic polyneuropathy in streptozotocin-induced type 1 diabetic rats

**DOI:** 10.3389/fncir.2014.00006

**Published:** 2014-01-29

**Authors:** Zhen-Zhen Kou, Chun-Yu Li, Jia-Chen Hu, Jun-Bin Yin, Dong-Liang Zhang, Yong-Hui Liao, Zhen-Yu Wu, Tan Ding, Juan Qu, Hui Li, Yun-Qing Li

**Affiliations:** Department of Anatomy, Histology and Embryology, K. K. Leung Brain Research Centre, The Fourth Military Medical UniversityXi‘an, China

**Keywords:** diabetic polyneuropathy, neural circuit, spinal cord, dorsal root ganglion, primary afferent, rat

## Abstract

Diabetic polyneuropathy (DPN) presents as a wide variety of sensorimotor symptoms and affects approximately 50% of diabetic patients. Changes in the neural circuits may occur in the early stages in diabetes and are implicated in the development of DPN. Therefore, we aimed to detect changes in the expression of isolectin B4 (IB4, the marker for nonpeptidergic unmyelinated fibers and their cell bodies) and calcitonin gene-related peptide (CGRP, the marker for peptidergic fibers and their cell bodies) in the dorsal root ganglion (DRG) and spinal cord of streptozotocin (STZ)-induced type 1 diabetic rats showing alterations in sensory and motor function. We also used cholera toxin B subunit (CTB) to show the morphological changes of the myelinated fibers and motor neurons. STZ-induced diabetic rats exhibited hyperglycemia, decreased body weight gain, mechanical allodynia and impaired locomotor activity. In the DRG and spinal dorsal horn, IB4-labeled structures decreased, but both CGRP immunostaining and CTB labeling increased from day 14 to day 28 in diabetic rats. In spinal ventral horn, CTB labeling decreased in motor neurons in diabetic rats. Treatment with intrathecal injection of insulin at the early stages of DPN could alleviate mechanical allodynia and impaired locomotor activity in diabetic rats. The results suggest that the alterations of the neural circuits between spinal nerve and spinal cord via the DRG and ventral root might be involved in DPN.

## Introduction

Diabetic polyneuropathy (DPN) is one of the most common complications of diabetes mellitus (DM; Dyck et al., 1993). DPN is not a single entity but encompasses several neuropathic syndromes, including sensory and motor defects (Yasuda et al et al., 2003; Boulton et al., 2005). Because DPN is rather complex and poorly understood and is most likely affected by multiple factors, diagnosis of DPN is seldom successful in the early to intermediate stages of DM (Tesfaye et al., 2010).

A large proportion of diabetic patients who suffer from DPN describe abnormal sensations such as pain in the early stages. Painful DPN in human subjects is usually characterized by the development of tactile allodynia, a condition in which light touch is perceived as painful (Baron et al., 2009). Tactile allodynia is observed in a large proportion (30–47%) of human subjects with DM (Bastyr et al., 2005). In contrast to sensory disturbance, motor function has rarely been studied in DPN (Andersen, 2012). However, previous reports demonstrate that type 1 diabetic patients exhibit impaired strength of the ankle and knee extensors and flexors, indicating that DPN is often a mixed sensory-motor neuropathy (Andersen, 2012; IJzerman et al., 2012). Despite considerable research on DPN, evidence for pathophysiological changes in the nervous systems underlying the impairment of motor function still lacks neurostructural evidence.

Previous studies have focused on the role of peripheral nerves in DPN. Increasing evidence now indicates that the insult of DPN might be at all levels of the nervous system, including both the PNS and the CNS (Eaton et al., 2001; Fischer and Waxman, 2010). Although the involvement of both PNS and CNS in DPN has previously been recognized, the underlying mechanisms remain poorly understood, particularly in the changes of the neural circuits between spinal nerves and spinal cord via the DRG and ventral root. Thus, investigating the alterations in the neural circuits is essential for understanding the development of the sensory and motor defects in DPN.

The spinal nerves conduct mixed signals, including sensory and motor information from nerve endings in the PNS (Todd, 2010). Spinal nerves have both dorsal and ventral roots. The dorsal root carries sensory axons originating from the sensory neurons in the dorsal root ganglion (DRG), while the ventral root carries motor axons originating from the motor neurons within the ventral horn of the spinal gray matter. The various types of DRG neurons send their central axonal processes to different parts of the spinal dorsal horn in the CNS (Duce and Keen, 1977; Todd, 2010). Three main types of nerve fibers originate in the DRG neurons and terminate in different laminae of the spinal dorsal horn: (1) unmyelinated non-peptidergic afferents are observed in laminae I and II; (2) peptidergic afferents are found in laminae I, II and V; and (3) myelinated afferents are located in laminae III and IV.

There are useful reagents for identifying and investigating the different nerve afferents. Cholera toxin B subunit (CTB) is selectively taken up by myelinated but not unmyelinated primary afferents (Tong et al., 1999; Shehab et al., 2004). When CTB is injected into sciatic nerves, it is selectively taken up by myelinated afferents and transported transganglionically to label fibers and terminals in laminae III to IV of the spinal dorsal horn and neurons in lamina IX of the ventral horn (Tong et al., 1999). For the unmyelinated afferents, the plant *Bandeiraea simplicifolia* I-isolectin B4 (IB4) binds to a subtype of small DRG neurons, specifically those that lack neuropeptides (Michael and Priestley, 1999). The antibody for calcitonin gene-related peptide (CGRP) recognizes small peptidergic neurons in the DRG and their afferents in spinal cord (Karanth et al., 1991).

It has been confirmed that DPN is irreversible when nerves are destroyed, so early intervention is very important to prevent neuropathic complications in patients with diabetes (Boulton et al., 2005; Tesfaye et al., 2010). Therefore, in the present study, we sought to clarify the changes of neural circuits at the early stages (within 4 weeks) of DPN. Using the model of streptozotocin (STZ)-induced type 1 diabetic rats, we examined the distributions and alterations of CTB-labeled myelinated, IB4-labled nonpeptidergic unmyelinated, and CGRP-immunopositive peptidergic fibers and their cell bodies in both DRG and spinal cord. We also applied insulin through intrathecal injection in diabetic rats to observe the effects of treatment on sensory and motor activities in behavioral tests.

## Materials and methods

### Experimental animals

All animal studies were conducted using approved protocols and carried out in accordance with the Principles of Laboratory Animal Care (NIH Publication no. 85-23, revised 1985). Male Sprague-Dawley rats weighing 220–250 g were obtained from the Laboratory Animal Center of The Fourth Military Medical University (Xi’an, China). In accordance with our previous studies (Zuo et al., 2011; Kou et al., 2013a), rats were injected with a single injection of 60 mg/kg STZ (Sigma, St. Louis, MO, USA), which was freshly dissolved in ice-cold sodium citrate (pH 4.5), while age-matched control rats received injections of a similar citrate buffer. Diabetes was confirmed on the third day by measurements of blood glucose concentrations in samples obtained from the tail vein using a strip-operated reflectance meter (Active; Roche Diagnostics, Mannheim, Germany). Only rats with blood glucose concentration >20 mM were used. All animals were housed in standard conditions (12 h light/dark cycles) with water and food available *ad libitum*.

### Pain behavioral test

The experiments were performed in accordance with our previously reported protocols (Mei et al., 2011; Kou et al., 2013a). To quantify the mechanical sensitivity of the hind paw, animals were placed in individual plastic boxes and allowed to acclimate for 30 min. A series of calibrated *von* Frey filaments (Stoelting, Kiel, WI, USA) ranging from 0.4 to 60.0 g were applied to the plantar surface of the hind paw, with sufficient force to bend the filaments for 5 s or until paw withdrawal. Applications were separated by 15 s intervals to allow the animal to cease any response and return to a relatively inactive position. In the presence of a response, the filament of the next lower force was applied. In the absence of a response, the filament of the next greater force was applied. A positive response was indicated by a sharp withdrawal of the paw. Each filament was applied 10 times, and the minimal value that caused at least six responses was recorded as the paw withdrawal threshold (PWT). All behavioral studies were performed under blind conditions.

### Open field test

An open field test was used to analyze the rats’ locomotor activity, as in our previous report (Quan-Xin et al., 2012). An animal was placed in one corner of the open field (100 × 100 × 48 cm). Movement of the rat in the area during the 15 min testing session was recorded. After 15 min, the rat was removed to the home cage, and the open field area was cleaned. The total distance and the average velocity in the area were measured.

### Immunohistochemistry

Rats were deeply anesthetized with the injection of pentobarbital (50 mg/kg, i.p.). All rats were perfused through the ascending aorta with 150 ml of 0.9% (w/v) saline followed by 50 ml of 4% (w/v) paraformaldehyde (Shanghai Xinran Biotechnology Co. Ltd.) and 0.2% (w/v) picric acid (Shanghai Xinran Biotechnology Co. Ltd.) in 0.1 M phosphate buffer (PB, pH 7.4) (Zuo et al., 2011; Kou et al., 2013a). After perfusion, lumbar segments of the spinal cord and the corresponding DRGs were removed, post-fixed with the same fresh fixative for 4 h and placed in 30% (w/v) sucrose solution (Shanghai Xinran Biotechnology Co. Ltd.) for 24 h at 4^°^C. The immunochemistry was performed as in our previous report (Kou et al., 2013b). Transverse sections of spinal cord (25 µm) and DRG (15 µm) were incubated in blocking solution (5% v/v normal goat serum) for 1 h at room temperature and then incubated overnight at 4^°^C with primary antibody: rabbit anti-CGRP (1:2000; Millipore, AB5920, CA, USA). The sections were then washed with 0.01 M phosphate buffered saline (PBS, pH 7.4) three times (10 min each). The sections were then incubated with Alexa594-conjugated donkey anti-rabbit IgG (1:1000; Invitrogen, A-21207, NY, USA) and fluorescein isothiocyanate (FITC)-IB4 (1:200; Vector Laboratories, FL-1201, CA, USA) for 2 h. Finally, the sections were rinsed with 0.01 M PBS, mounted onto clean glass slides, air-dried and cover slipped with a mixture of 0.05 M PBS containing 50% (v/v) glycerin and 2.5% (w/v) triethylenediamine. The sections were observed under a laser-scanning confocal microscope (FV-1000, Olympus, Tokyo, Japan).

### Neurotracer injection

The rats were deeply anesthetized by injection of pentobarbital, and the sciatic nerves on the left side were exposed. Then, 2 µl of 1% Alexa594-conjugated CTB (red fluorescence, Molecular Probes, C22842, CA, USA) was slowly injected with a 33-gauge Hamilton syringe into the proximal part of the sciatic nerve after it was exposed at the mid-thigh level. The incision was closed by suture. Three days after CTB injection, the rats were perfused. DRGs and lumbar segments were removed and processed as described for immunohistochemistry.

### Intrathecal injection

Intrathecal implantation was performed as in our previous reports (Mei et al., 2011; Kou et al., 2013a). A polyethylene (PE) tube (Intramedic^®^, Becton Dickinson and Company, NJ, USA) was inserted directly into the subarachnoid space of the lumbar enlargement (between L4 to L6) for drug injection. Under pentobarbital anesthesia, a midline incision from L6 to L3 was made along the back of the rat. A pre-measured length of PE-10 tube (I.D. 0.28 and O.D. 0.61 mm) was passed caudally into the lumbar enlargement at the level of the lumbar vertebra. Only rats judged to be neurologically normal and showing complete paralysis of the tail and bilateral hind legs after administration of 2% lidocaine (10 µl) intrathecally were used for the following experiments. After tube implantation, rats were allowed to recover for 2 days. The rats were intrathecally injected with either insulin (0.2 units) or saline (10 µl) through the catheter at 10:00–10:30 am every day for 14 days (Figure 5).

### Statistical analyses

Immunohistochemical data were analyzed in accordance with previous reports (Fernyhough et al., 1989; Aizawa and Eggermont, 2006; Mei et al., 2011; Zuo et al., 2011; Kou et al., 2013a; Kou et al., 2013a,b). For quantification of immunopositive cell profiles in the DRG and the spinal ventral horn, five sections from a series of every fourth serial section of the lumbar segments (L4–L6) of spinal cord (25 µm) and DRG (15 µm) were selected randomly from each animal. In each group, six rats were used for statistical analysis. All positively stained cells in the area were evaluated with ImageJ software (National Institutes of Health).^1^ Statistical analysis was performed with one-way ANOVA with the Student–Newman–Kuels (SNK) *post hoc* test, using SPSS 16.0 (SPSS Inc., Chicago, IL, USA). The changes in the small neurons among the total CTB-positive neurons in the DRG were analyzed by Cross-Tabulation with significance testing with Pearson’s Chi-square. For analysis of immunopositive terminals in the spinal dorsal horn, the relative optical densities (ROD) of the immunostaining were analyzed with ImageJ software (Burgess et al., 2010). First, the integrated optical density (IOD) of the positive staining and background were acquired from five sections from each rat. Then, the ROD was calculated by subtracting the background from the IOD of the positive staining. The values for ROD were statistically analyzed among groups with SPSS. Data from immunofluorescence were expressed as fold change vs. the control group.

Analysis of the time-course of the behavioral tests between the saline- and insulin-treated groups was performed by two-factor (group and times) repeated measures analysis of variance (ANOVA). One-way ANOVA was employed for analyzing other data and was followed by the least significant difference (LSD) (equal variances assumed) or Dennett’s T3 (equal variances not assumed) *post hoc* test. Data were expressed as the mean ± SD. Differences between groups were considered statistically significant at a value of *p* < 0.05.

## Results

### Streptozotocin (STZ)-induced type 1 diabetic rat

**Figure 1:**
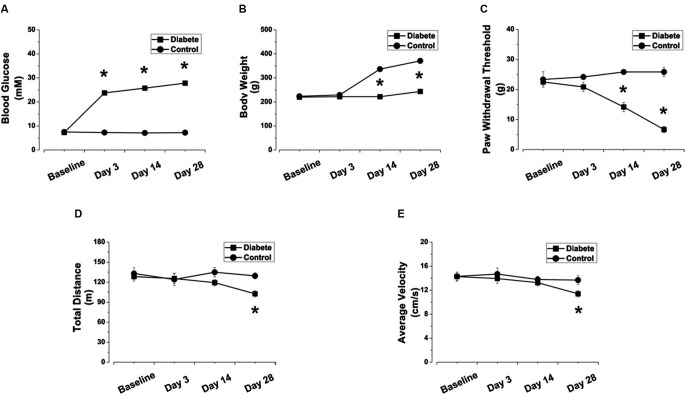
**STZ-induced diabetic rats exhibited elevated blood glucose concentration, reduced body weight gain, decreased PWTs, and impaired locomotor activity.** Changes in blood glucose concentration **(A)**, body weight **(B)**, PWT **(C)**, total distance **(D)** and average velocity **(E)** in STZ-induced diabetic rats. Mean ± SD, *n* = 6–18 rats in each group. * *P* < 0.05, comparing diabetic rats with non-diabetic control rats.

Following a single *i.p.* injection of STZ (60 mg/kg), we monitored rats’ blood glucose and body weight for 28 days. No difference in basal blood glucose concentration and body weight existed between STZ-treated rats and the control rats at the onset of the study (Figure 1A). However, compared with control rats, rats treated with STZ showed significant hyperglycemia at day 3 (23.75 ± 0.87 vs. 7.27 ± 0.30 mM in the control group, *p* < 0.05), which was maintained until day 28 (Figure 1A). Fourteen days after the onset of diabetes, body weight in the diabetic rats was significantly lower than in the control group (221.67 ± 6.00 vs. 336.67 ± 6.15 g in the control group, *p* < 0.05) and continued lower thereafter until day 28 (Figure 1B). These data show that rats injected with STZ developed type 1 diabetic features such as hyperglycemia and reduced body weight gain.

### Mechanical allodynia and impaired locomotor activity in streptozotocin (STZ)-induced type 1 diabetic rats

Fourteen days after STZ injection, the PWTs in diabetic rats were significantly lower than those in control rats when tested with *von* Frey filaments (14.17 ± 1.54 vs. 25.83 ± 0.83 g in the control group,* p* < 0.05, Figure 1C) and were further reduced at day 28 (6.67 ± 1.05 vs. 25.83 ± 1.54 g in the control group, *p* < 0.05, Figure 1C). The results indicate that, along with elevated blood glucose levels, the diabetic rats increasingly developed mechanical allodynia during days 14–28 (Figure 1C).

To determine whether STZ-induced diabetic rats also exhibited impaired locomotor activity, we assessed the total travel distance and average velocity in the open field test (Figures 1D, E). Significant reductions in total distance (102.54 ± 4.70 vs. 128.39 ± 6.68 m in control, *p* < 0.05, Figure 1D) and average velocity (11.39 ± 0.52 vs. 14.27 ± 0.74 cm/s in control, *p* < 0.05, Figure 1E) were observed in diabetic rats at day 28.

### Changes in isolectin B4 (IB4)-labeled and calcitonin gene-related peptide (CGRP)-immunoreactive structures in the dorsal root ganglion (DRG) and spinal dorsal horn

**Figure 2:**
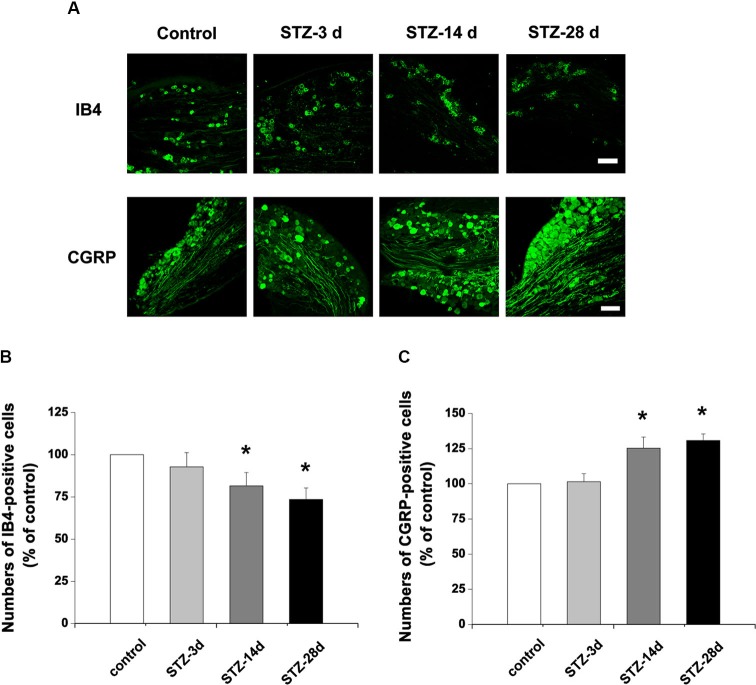
**Photographs showing IB4- and CGRP-stained neurons in the DRG. (A)** Scale bar = 100 µm. Statistical analyses of the numbers of IB4-labeled **(B)** and CGRP-immunoreactive (IR) neurons **(C)** in different groups. Values are normalized, with the mean of the control group set as 100%. Mean ± SD, *n* = 6 rats in each group. * *P* < 0.05, comparing diabetic rats with non-diabetic control rats.

**Figure 3:**
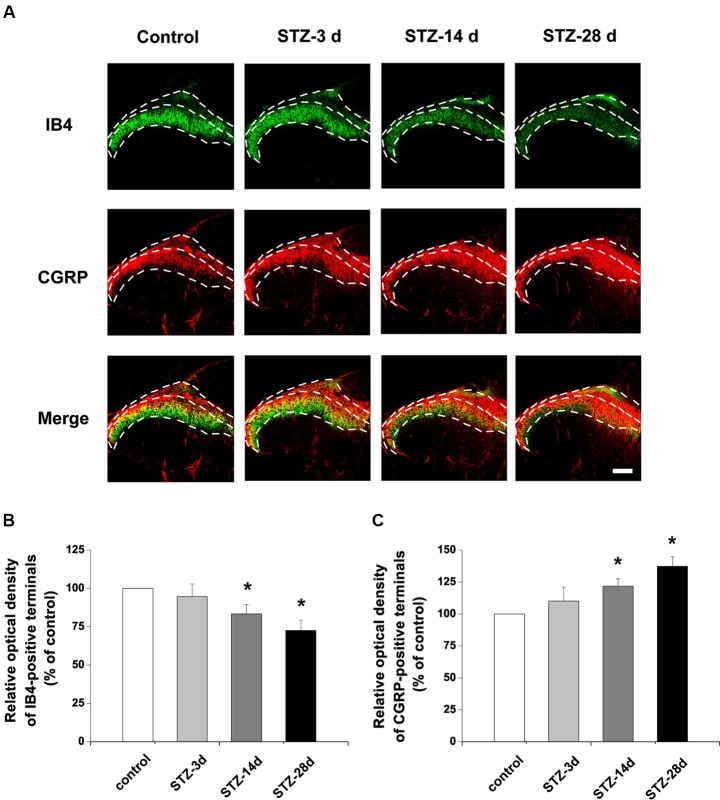
**Distribution and expression changes of IB4 labeling and CGRP immunoreactivity are observed in the spinal dorsal horn.** Double fluorescent labeling for IB4 (green) and CGRP (red) shows that the expression of CGRP increased but IB4 staining decreased in diabetic rats **(A)**. Scale bar = 100 µm. Statistical analyses of the expression of IB4 **(B)** and CGRP **(C)** in different groups. Values are normalized, with the mean of the control group set as 100%. Mean ± SD, *n* = 6 rats in each group. * *P* < 0.05, comparing diabetic rats with non-diabetic control rats.

To investigate whether the nonpeptidergic unmyelinated fibers and their cell bodies, and the peptidergic fibers and their cell bodies are involved in DPN, we examined the IB4-labeled and CGRP-immunoreactive (IR) structures in the DRG and spinal dorsal horn. In DRG, IB4-labeled and CGRP-IR cell bodies are small (diameter ≤20 µm) to medium (20 µm < diameter ≤35 µm) neurons (Wang et al., 2010). Compared with controls, the number of IB4-labeled neurons decreased from day 14 (81.5% of control) to day 28 (73.5% of control) after onset of diabetes (*p* < 0.05, Figures 2A, B). The number of CGRP-IR neurons, however, significantly increased, measuring 125.3% of control at day 14 and 130.8% of control at day 28 (*p* < 0.05, Figures 2A, C). Similar changes occurred in the superficial laminae of the spinal dorsal horn, with IB4-labeled terminals decreasing (day 14, 83.4% of control, *p* < 0.05; day 28, 72.6% of control, *p* < 0.05, Figures 3A, B). CGRP-IR terminals were densely concentrated and increased in the inner part of lamina II (*p* < 0.05, Figures 3A, C). These data demonstrate that in both DRG and spinal dorsal horn, compared with the controls, IB4-labeled structures decreased and CGRP-IR structures increased in diabetic rats.

### Changes of cholera toxin B (CTB)-labeled structures in the dorsal root ganglion (DRG) and spinal cord

**Figure 4:**
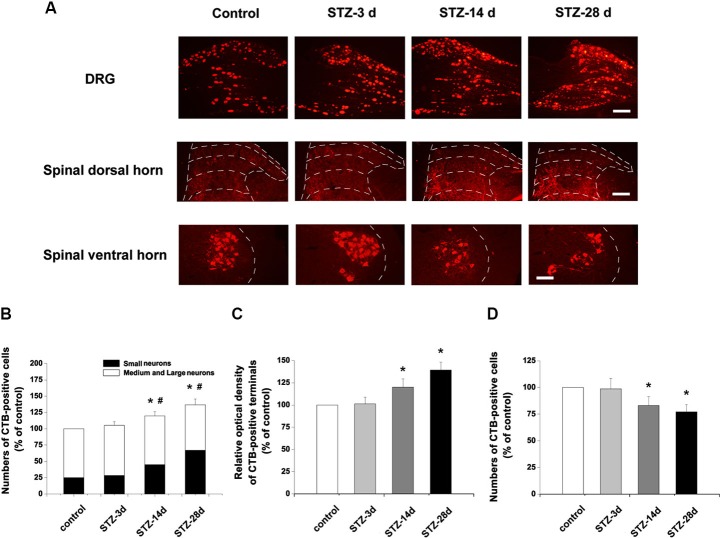
**Changes of CTB-labeled elements in the DRG and spinal dorsal and ventral horns. (A)** Scale bar = 100 µm. Statistical analyses of CTB labeling in the DRG **(B)**, spinal dorsal horn **(C)** and spinal ventral horn **(D)**. Values are normalized, with the mean of the control group set as 100%. The proportions of small neurons to the total CTB-labeled DRG neurons are 25.2% in the control, 27.1% in STZ-3d, 37.7% in STZ-14d and 46.1% in STZ-28d groups. Mean ± SD, *n* = 6 rats for each group. * *P* < 0.05, comparing the numbers of CTB-labeled cells in diabetic rats with those in non-diabetic control rats. ^#^ *P* < 0.05, comparing the proportions of the small CTB-labeled DRG neurons in diabetic rats with non-diabetic control rats.

Consistent with a previous report (Wang et al., 2010), CTB-labeled elements were mainly large (diameter > 35 µm) neurons in controls. Numbers of CTB-labeled neurons markedly increased in the DRG, up to 119.8% of control at day 14 and 136.8% of control at day 28 (*p* < 0.05, Figures 4A, B). Notably, there were more small DRG neurons labeled with CTB in diabetic rats. In controls, 25.2% of CTB-labeled neurons were small neurons. However, the small CTB-labeled neurons increased in number from day 14 to day 28 in STZ-treated rats (*p* < 0.05, Figures 4A, B).

In spinal cord, CTB-labeling is present in both the dorsal and ventral horns of the spinal cord (Figure 4A). In the dorsal horn, CTB-labeled terminals were densely located in laminae III to IV, with some labeling in laminae I and II. In diabetic rats, CTB immunoreactivity was significantly increased at day 14 (120.1% of control,* p* < 0.05) and at day 28 (139.3% of control, *p* < 0.05, Figures 4A, C). Importantly, compared with the controls, the diabetic rats showed denser immunoreactivity for CTB in the laminae III to IV of the spinal dorsal horn (Figure 4A). Numbers of CTB-labeled motor neurons in the ventral horn decreased significantly in diabetic rats (day 14, 83.2% of control, *p* < 0.05; day 28, 77.2% of control, *p* < 0.05, Figures 4A, D).

### Effects of intrathecal injection of insulin on pain behavior and locomotor activity

**Figure 5:**
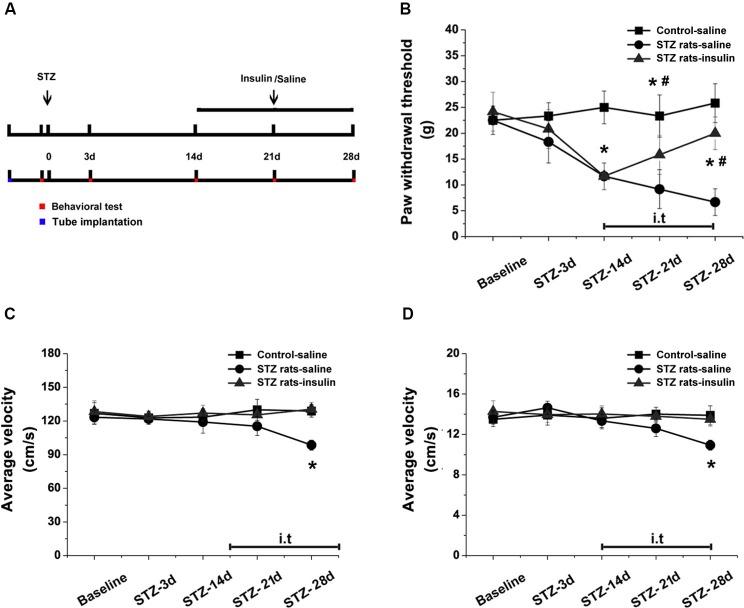
**Effects of intrathecal insulin on pain behavior and locomotor activity in diabetic rats compared with controls.** PWTs and open field tests were measured before (baseline) and 3, 14, 21 and 28 days after STZ injection, with and without insulin application from day 14 to day 28 **(A)**. Intrathecal injection of insulin alleviated mechanical allodynia **(B)** and impaired locomotor activity (total distance, **(C)**, and average velocity, **(D)**, in the open field test) in diabetic rats after 14 days of administration. Mean ± SD, *n* = 6 rats in each group. * *P* < 0.05, comparing the STZ rats-insulin group with the STZ rats-saline group at corresponding time points. ^#^ *P* < 0.05, comparing the STZ rats-insulin group with the control-saline group at corresponding time points.

To detect the effects of insulin at the early stages of DPN, either saline or insulin (0.2 units) was injected intrathecally once a day from day 14 to day 28 in STZ-induced diabetic rats. PWTs were tested on the day of STZ injection and at 3, 14, 21, and 28 days post-STZ injection (Figure 5A). Intrathecal injection of insulin significantly alleviated allodynia in diabetic rats after 14 days (*p* < 0.05 vs. STZ rats-saline group), but could not completely attenuate the reduced PWTs (*p* < 0.05 vs. wild type-saline group, Figure 5B). Fourteen days of intrathecal insulin injection also prevented the decrease in total distance (*p* < 0.05 vs. STZ rats-saline group, *p* > 0.05 vs. wild type-saline group, Figure 5C) and average velocity (*p* < 0.05 vs. STZ rats-saline group, *p* > 0.05 vs. wild type-saline group, Figure 5D) in diabetic rats. These data indicate that insulin treatment at the early stages of DPN reduced the mechanical allodynia and impaired locomotor activity normally present in STZ-induced diabetic rats.

## Discussion

STZ-induced diabetic rodents are widely used for studying DPN (Mitsuhashi et al., 1993; Brussee et al., 2004; Zuo et al., 2011; Zguira et al., 2013). Our results show that STZ-induced diabetic rats exhibit hyperglycemia, decreased body weight gain and mechanical allodynia after STZ injection, as well as impaired locomotor activity at day 28, supporting the use of STZ-induced diabetic rats as an animal model for studying the mechanism of the early stages of type 1 DPN.

### Changes in isolectin B4 (IB4)-, calcitonin gene-related peptide (CGRP)- and cholera toxin B (CTB)-labeled neurons in the dorsal root ganglion (DRG)

DRG neurons receive a wide range of sensory information from nerve endings that are activated by mechanical, thermal, chemical and noxious stimuli (Delmas et al., 2011; Hughes et al., 2012). Nerve injury can induce marked changes in the expression of neuropeptides, neuromodulators, channels and related receptors in DRG neurons, resulting in a virtually new phenotype of DRG neurons (Hokfelt et al., 2000; Liu et al., 2000; Lai et al., 2004). For instance, after peripheral nerve injury, reduced IB4 binding was observed (Molander et al., 1996; Bennett et al., 1998). Importantly, in diabetic patients with DPN, there have been reports of abnormal function of unmyelinated afferents, suggesting that impairment in the receptive properties of unmyelinated nerves leads to DPN (Orstavik et al., 2006). Our results indicate that in DRG of diabetic rats, the number of IB4-labeled neurons decreased significantly from day 14, suggesting that decreased nonpeptidergic unmyelinated afferents might be one reason for early sensory dysfunction in diabetes.

In contrast to IB4, the number of CGRP-IR neurons increased from day 14 to day 28. It has been confirmed that CGRP is a pain-related peptide and serves as a pain modulator, contributing to the augmented synaptic strength between DRG neurons and the spinal dorsal horn and leading to the generation of central sensitization in pain (Sun et al., 2003; Bird et al., 2006). Following peripheral inflammation or partial nerve injury, CGRP may be up-regulated and thus contribute to the generation of pain (Fehrenbacher et al., 2003; Scholz and Woolf, 2007). Our results demonstrate that CGRP expression increased in DRG neurons of diabetic rats from day 14 onward, suggesting that a subpopulation of peptidergic afferents might also be involved in painful DPN. Previous reports demonstrate that the CGRP expression in STZ-induced diabetic rodents either remains unchanged or decreases after 4 weeks (Diemel et al., 1994; Akkina et al., 2001). It is known that early intervention is very important for treating neuropathic complications in diabetes (Boulton et al., 2005), and rats treated with STZ developed mechanical allodynia beginning on day 14; therefore, we focused on the changes in neural circuits in the early stages (within 4 weeks). CGRP is a nerve growth factor (NGF)-dependent neuropeptide. In the early stages, the NGF production by DRG neurons increases in STZ-treated diabetic rats, but it gradually decreases after 4 weeks (Steinbacher and Nadelhaft, 1998). With the decreased support from NGF after 4 weeks, the expression of CGRP may decrease. The activation of transient receptor potential vanilloid subtype 1 (TRPV1) channel leads to the release of CGRP (Huang et al., 2008). The expression and function of TRPV1 is initially enhanced but is reduced in the later phase in diabetic rats (Pabbidi et al., 2008). Previous report also indicates similar changes in the early and late stages of human diabetes (Dyck et al., 2000). STZ-induced diabetes also shows two phases of pain sensitivity (Pabbidi et al., 2008). Therefore, our results suggest that the increased CGRP expression from day 14 to 28 might indicate the early changes in DPN. However, we could not exclude the possibility that other factors might be involved. For instance, differences in CGRP innervation in diabetic rats and mice have been demonstrated (Karanth et al., 1990; Christianson et al., 2003). Cutaneous CGRP-IR nerves are largely lost in diabetic mice, whereas they increase in STZ-induced diabetic rats. Further, the variations in the dosage of STZ and in the initial age of animals used in research, both of which are important for the development of diabetes, might also contribute to the differences among reports.

In diabetic rats, there was also a significant increase in the number of CTB-labeled neurons in the DRG. In normal rats, CTB-labeled cells are mainly large DRG neurons that send myelinated fibers to the spinal dorsal horn and are involved in proprioception (Tong et al., 1999; Shehab et al., 2004). Interestingly, in diabetic rats, CTB labeling was also found in many small neurons, indicating a shift in tracer uptake in small DRG neurons in diabetes. A similar change in CTB-labeling phenotype has been demonstrated in nerve injury (Tong et al., 1999). Therefore, these phenotypic changes in these subpopulations of DRG neurons might contribute to the sensory dysfunction in the early stages of DPN.

### Changes in isolectin B4 (IB4), calcitonin gene-related peptide (CGRP) and cholera toxin B (CTB) labeling in the spinal dorsal horn

It has been shown that nociceptive signals are conveyed from the DRG to the CNS for integration, beginning in the spinal cord (Todd, 2010). Thus, the afferents in the spinal cord from the DRG are essential for understanding the mechanisms of DPN in the CNS. Our data also show that afferents in the dorsal horn, which showed IB4 labeling and CGRP immunoreactivity, were altered in diabetic rats. IB4 immunoreactivity decreased, whereas CGRP-positive terminals increased. Increased production of CGRP from primary sensory neurons is also present in other pathological pain conditions (Fehrenbacher et al., 2003; Scholz and Woolf, 2007). Therefore, the changes in IB4-labeled and CGRP-IR central axons originating from DRG neurons in diabetic rats might also contribute to painful DPN in the spinal dorsal horn. We also found that CTB-positive nerve endings increased in laminae III and IV in diabetic rats, and there was also nerve-injury-induced sprouting of myelinated afferent fibers from deeper laminae. (Tong et al., 1999; Tan et al., 2012). In addition, it is known that myelinated fibers normally respond to innocuous sensations such as touch and pressure (Woolf, 1995). Therefore, the reorganization of such structures in diabetes may contribute to the sustained pain and mechanical allodynia in diabetic rats. Combined with the data showing that more small DRG neurons in diabetic rats express CTB, it is possible that, at least in part, the increased sprouting of CTB-labeled fibers in diabetic rats is attributable to phenotypic changes in axons, resulting in the axons from small DRG neurons in spinal dorsal horn becoming CTB-positive. Moreover, the complicated physiological conditions displayed by diabetic rats, including hyperglycemia and hypoxia, might be possible reasons for the increased sprouting of myelinated terminals in the spinal dorsal horn. Thus, the changes in neural circuits from the peripheral nerves to the spinal dorsal horn might be involved in the progressive sensory defects in DPN.

### Changes of cholera toxin B (CTB)-labeled neurons in the spinal ventral horn

Motor diabetic neuropathy is a prominent complication in diabetes (Andersen and Mogensen, 1997; Andersen, 2012). Decline in nerve conduction velocity and motor function impairment are evident during early stages of diabetes in human patients (Dyck et al., 1986; Dyck and Giannini, 1996; Malik et al., 2001). The open field test data presented here show that locomotor activity, including total distance and average velocity, decreased at day 28 in diabetic rats. However, mechanical allodynia may also be impairing locomotor activity in diabetic rats. To clarify this issue, we injected CTB into the sciatic nerve to simultaneously observe the transportation of the neurotracer from the sciatic nerve to the spinal dorsal and ventral horns. This method could show the changes in sensory and motor regions at the same time points. CTB labeling in motor neurons decreased beginning on day 14 in diabetic rats, indicating that structural abnormalities occur in the CNS before motor dysfunction presents. These changes in the motor neural circuit from the peripheral nerves to the spinal ventral horn might lead to impaired motor activity in the early stages of DPN independent of mechanical allodynia.

A previous report demonstrates that motor neurons develop progressive features of distal loss of axonal terminals in chronic STZ-induced diabetic rats (Ramji et al., 2007). Although motor impairment might begin later than sensory defects in diabetes (Dyck et al., 1986; Dyck and Giannini, 1996; Malik et al., 2001), declines in motor nerve conduction velocity are evident beginning at 2 weeks in diabetic rats (Coppey et al., 2000), which is consistent with our results showing decreased CTB labeling in the spinal ventral horn on day 14. Therefore, the reduced CTB labeling at the early stages might suggest a defect in the transportation of CTB through the ventral root of the spinal nerve. However, because of the different neural structure, the blood-brain barrier might provide greater protection for the motor neurons in the spinal ventral horn than for sensory neurons in the DRG. The underlying relationship between the impaired motor activity and motor neural circuit requires further investigation.

### Intrathecal injection of insulin alleviates mechanical allodynia and impaired locomotor activity

Because STZ-induced diabetic rats are insulin deficient, it was important to test whether insulin treatment would help to maintain normal neurotransmission, particularly because physiological concentrations of insulin directly enhance axon formation in the DRG and spinal cord (Fernyhough et al., 1993; Huang et al., 2005). Moreover, insulin modulates neurofilament and tubulin abundance, which is correlated with the induction of neurite elongation (Fernyhough et al., 1989). We therefore injected insulin for 14 days. Intrathecal injection of insulin significantly alleviated mechanical allodynia and impaired locomotor activity in diabetic rats, indicating that early intervention is important for treating DPN.

In summary, we have described the changes in the neural circuits in STZ-induced diabetic rats with progressive mechanical allodynia and impaired locomotor activity. The alterations in myelinated nerve fibers, unmyelinated nonpeptidergic nerve fibers, and peptidergic nerve fibers might be involved in the early stages of the development in DPN. The underlying mechanism of DPN might be addressed by the dysfunction of those subpopulations of afferents from the PNS to the CNS.

## Conflict of interest statement

The authors declare that the research was conducted in the absence of any commercial or financial relationships that could be construed as a potential conflict of interest.

## Footnotes

^1^http://rsbweb.nih.gov/ij/

## References

[B1] Aizawa N. Eggermont J. J. (2006). Effects of noise-induced hearing loss at young age on voice onset time and gap-in-noise representations in adult cat primary auditory cortex. J. Assoc. Res. Otolaryngol. 7, 71–81 10.1007/s10162-005-0026-3 16408166 PMC2504589

[B2] Akkina S. K. Patterson C. L. Wright D. E. (2001). GDNF rescues nonpeptidergic unmyelinated primary afferents in streptozotocin-treated diabetic mice. Exp. Neurol. 167, 173–182 10.1006/exnr.2000.7547 11161605

[B3] Andersen H. (2012). Motor dysfunction in diabetes. Diabetes Metab. Res. Rev. 28(Suppl. 1), 89–92 10.1002/dmrr.2257 22271730

[B4] Andersen H. Mogensen P. H. (1997). Disordered mobility of large joints in association with neuropathy in patients with long-standing insulin-dependent diabetes mellitus. Diabet. Med. 14, 221–227 10.1002/(sici)1096-9136(199703)14:3<221::aid-dia338>3.0.co;2-k 9088771

[B5] Baron R. Tolle T. R. Gockel U. Brosz M. Freynhagen R. (2009). A cross-sectional cohort survey in 2100 patients with painful diabetic neuropathy and postherpetic neuralgia: differences in demographic data and sensory symptoms. Pain 146, 34–40 10.1016/j.pain.2009.06.001 19592166

[B6] Bastyr E. J. 3rd Price K. L. Bril V. (2005). Development and validity testing of the neuropathy total symptom score-6: questionnaire for the study of sensory symptoms of diabetic peripheral neuropathy. Clin. Ther. 27, 1278–1294 10.1016/j.clinthera.2005.08.002 16199253

[B7] Bennett D. L. Michael G. J. Ramachandran N. Munson J. B. Averill S. Yan Q. (1998). A distinct subgroup of small DRG cells express GDNF receptor components and GDNF is protective for these neurons after nerve injury. J. Neurosci. 18, 3059–3072 9526023 10.1523/JNEUROSCI.18-08-03059.1998 PMC6792585

[B8] Bird G. C. Han J. S. Fu Y. Adwanikar H. Willis W. D. Neugebauer V. (2006). Pain-related synaptic plasticity in spinal dorsal horn neurons: role of CGRP. Mol. Pain 2:31 10.1186/1744-8069-2-31 17002803 PMC1592081

[B9] Boulton A. J. Vinik A. I. Arezzo J. C. Bril V. Feldman E. L. Freeman R. (2005). Diabetic neuropathies: a statement by the American Diabetes Association. Diabetes Care 28, 956–962 10.2337/diacare.28.4.956 15793206

[B10] Brussee V. Cunningham F. A. Zochodne D. W. (2004). Direct insulin signaling of neurons reverses diabetic neuropathy. Diabetes 53, 1824–1830 10.2337/diabetes.53.7.1824 15220207

[B11] Burgess A. Vigneron S. Brioudes E. Labbe J. C. Lorca T. Castro A. (2010). Loss of human greatwall results in G2 arrest and multiple mitotic defects due to deregulation of the cyclin B-Cdc2/PP2A balance. Proc. Natl. Acad. Sci. U S A 107, 12564–12569 10.1073/pnas.0914191107 20538976 PMC2906566

[B12] Christianson J. A. Riekhof J. T. Wright D. E. (2003). Restorative effects of neurotrophin treatment on diabetes-induced cutaneous axon loss in mice. Exp. Neurol. 179, 188–199 10.1016/s0014-4886(02)00017-1 12618126

[B13] Coppey L. J. Davidson E. P. Dunlap J. A. Lund D. D. Yorek M. A. (2000). Slowing of motor nerve conduction velocity in streptozotocin-induced diabetic rats is preceded by impaired vasodilation in arterioles that overlie the sciatic nerve. Int. J. Exp. Diabetes Res. 1, 131–143 10.1155/edr.2000.131 11469397 PMC2477757

[B14] Delmas P. Hao J. Rodat-Despoix L. (2011). Molecular mechanisms of mechanotransduction in mammalian sensory neurons. Nat. Rev. Neurosci. 12, 139–153 10.1038/nrn2993 21304548

[B15] Diemel L. T. Brewster W. J. Fernyhough P. Tomlinson D. R. (1994). Expression of neuropeptides in experimental diabetes; effects of treatment with nerve growth factor or brain-derived neurotrophic factor. Brain Res. Mol. Brain Res. 21, 171–175 10.1016/0169-328x(94)90391-3 7513041

[B16] Duce I. R. Keen P. (1977). An ultrastructural classification of the neuronal cell bodies of the rat dorsal root ganglion using zinc iodide-osmium impregnation. Cell Tissue Res. 185, 263–277 10.1007/bf00220670 597845

[B17] Dyck P. J. Giannini C. (1996). Pathologic alterations in the diabetic neuropathies of humans: a review. J. Neuropathol. Exp. Neurol. 55, 1181–1193 10.1097/00005072-199612000-00001 8957441

[B18] Dyck P. J. Kratz K. M. Karnes J. L. Litchy W. J. Klein R. Pach J. M. (1993). The prevalence by staged severity of various types of diabetic neuropathy, retinopathy and nephropathy in a population-based cohort: the Rochester Diabetic Neuropathy Study. Neurology 43, 817–824 10.1212/wnl.43.4.817 8469345

[B19] Dyck P. J. Lais A. Karnes J. L. O’brien P. Rizza R. (1986). Fiber loss is primary and multifocal in sural nerves in diabetic polyneuropathy. Ann. Neurol. 19, 425–439 10.1002/ana.410190503 3717906

[B20] Dyck P. J. Larson T. S. O’brien P. C. Velosa J. A. (2000). Patterns of quantitative sensation testing of hypoesthesia and hyperalgesia are predictive of diabetic polyneuropathy: a study of three cohorts. Nerve growth factor study group. Diabetes Care 23, 510–517 10.2337/diacare.23.4.510 10857944

[B21] Eaton S. E. Harris N. D. Rajbhandari S. M. Greenwood P. Wilkinson I. D. Ward J. D. (2001). Spinal-cord involvement in diabetic peripheral neuropathy. Lancet 358, 35–36 10.1016/S0140-6736(00)05268-5 11454377

[B22] Fehrenbacher J. C. Taylor C. P. Vasko M. R. (2003). Pregabalin and gabapentin reduce release of substance P and CGRP from rat spinal tissues only after inflammation or activation of protein kinase C. Pain 105, 133–141 10.1016/s0304-3959(03)00173-8 14499429

[B23] Fernyhough P. Mill J. F. Roberts J. L. Ishii D. N. (1989). Stabilization of tubulin mRNAs by insulin and insulin-like growth factor I during neurite formation. Brain Res. Mol. Brain Res. 6, 109–120 10.1016/0169-328x(89)90044-2 2693875

[B24] Fernyhough P. Willars G. B. Lindsay R. M. Tomlinson D. R. (1993). Insulin and insulin-like growth factor I enhance regeneration in cultured adult rat sensory neurones. Brain Res. 607, 117–124 10.1016/0006-8993(93)91496-f 8481790

[B25] Fischer T. Z. Waxman S. G. (2010). Neuropathic pain in diabetes—evidence for a central mechanism. Nat. Rev. Neurol. 6, 462–466 10.1038/nrneurol.2010.90 20625378

[B26] Hokfelt T. Broberger C. Xu Z. Q. Sergeyev V. Ubink R. Diez M. (2000). Neuropeptides–an overview. Neuropharmacology 39, 1337–1356 10.1016/s0028-3908(00)00010-1 10818251

[B27] Huang T. J. Verkhratsky A. Fernyhough P. (2005). Insulin enhances mitochondrial inner membrane potential and increases ATP levels through phosphoinositide 3-kinase in adult sensory neurons. Mol. Cell. Neurosci. 28, 42–54 10.1016/j.mcn.2004.08.009 15607940

[B28] Huang W. Wang H. Galligan J. J. Wang D. H. (2008). Transient receptor potential vanilloid subtype 1 channel mediated neuropeptide secretion and depressor effects: role of endoplasmic reticulum associated Ca2+ release receptors in rat dorsal root ganglion neurons. J. Hypertens. 26, 1966–1975 10.1097/hjh.0b013e328309eff9 18806620 PMC2669742

[B29] Hughes J. P. Chessell I. Malamut R. Perkins M. Backonja M. Baron R. (2012). Understanding chronic inflammatory and neuropathic pain. Ann. N Y Acad. Sci. 1255, 30–44 10.1111/j.1749-6632.2012.06561.x 22564068

[B30] Karanth S. S. Springall D. R. Francavilla S. Mirrlees D. J. Polak J. M. (1990). Early increase in CGRP- and VIP-immunoreactive nerves in the skin of streptozotocin-induced diabetic rats. Histochemistry 94, 659–666 10.1007/bf00271994 1704001

[B31] Karanth S. S. Springall D. R. Kuhn D. M. Levene M. M. Polak J. M. (1991). An immunocytochemical study of cutaneous innervation and the distribution of neuropeptides and protein gene product 9.5 in man and commonly employed laboratory animals. Am. J. Anat. 191, 369–383 10.1002/aja.1001910404 1719791

[B32] Kou Z. Z. Li C. Y. Tang J. Hu J. C. Qu J. Liao Y. H. (2013a). Down-regulation of insulin signaling is involved in painful diabetic neuropathy in type 2 diabetes. Pain Physician 16, E71–E83 23511693

[B33] Kou Z. Z. Qu J. Zhang D. L. Li H. Li Y. Q. (2013b). Noise-induced hearing loss is correlated with alterations in the expression of GABAB receptors and PKC gamma in the murine cochlear nucleus complex. Front. Neuroanat. 7:25 10.3389/fnana.2013.00025 23908607 PMC3726868

[B34] Lai J. Porreca F. Hunter J. C. Gold M. S. (2004). Voltage-gated sodium channels and hyperalgesia. Annu. Rev. Pharmacol. Toxicol. 44, 371–397 10.1146/annurev.pharmtox.44.101802.121627 14744251

[B35] Liu C. N. Wall P. D. Ben-Dor E. Michaelis M. Amir R. Devor M. (2000). Tactile allodynia in the absence of C-fiber activation: altered firing properties of DRG neurons following spinal nerve injury. Pain 85, 503–521 10.1016/s0304-3959(00)00251-7 10781925

[B36] Malik R. A. Veves A. Walker D. Siddique I. Lye R. H. Schady W. (2001). Sural nerve fibre pathology in diabetic patients with mild neuropathy: relationship to pain, quantitative sensory testing and peripheral nerve electrophysiology. Acta Neuropathol. 101, 367–374 11355308 10.1007/s004010000287

[B37] Mei X. P. Zhou Y. Wang W. Tang J. Zhang H. Xu L. X. (2011). Ketamine depresses toll-like receptor 3 signaling in spinal microglia in a rat model of neuropathic pain. Neurosignals 19, 44–53 10.1159/000324293 21389680

[B38] Michael G. J. Priestley J. V. (1999). Differential expression of the mRNA for the vanilloid receptor subtype 1 in cells of the adult rat dorsal root and nodose ganglia and its downregulation by axotomy. J. Neurosci. 19, 1844–1854 10024368 10.1523/JNEUROSCI.19-05-01844.1999 PMC6782176

[B39] Mitsuhashi T. Nakayama H. Itoh T. Kuwajima S. Aoki S. Atsumi T. (1993). Immunochemical detection of advanced glycation end products in renal cortex from STZ-induced diabetic rat. Diabetes 42, 826–832 10.2337/diabetes.42.6.826 8495806

[B40] Molander C. Wang H. F. Rivero-Melian C. Grant G. (1996). Early decline and late restoration of spinal cord binding and transganglionic transport of isolectin B4 from Griffonia simplicifolia I after peripheral nerve transection or crush. Restor. Neurol. Neurosci. 10, 123–133 10.3233/RNN-1996-10301 21551513

[B41] Orstavik K. Namer B. Schmidt R. Schmelz M. Hilliges M. Weidner C. (2006). Abnormal function of C-fibers in patients with diabetic neuropathy. J. Neurosci. 26, 11287–11294 10.1523/jneurosci.2659-06.2006 17079656 PMC6674548

[B42] Pabbidi R. M. Yu S. Q. Peng S. Khardori R. Pauza M. E. Premkumar L. S. (2008). Influence of TRPV1 on diabetes-induced alterations in thermal pain sensitivity. Mol. Pain 4:9 10.1186/1744-8069-4-9 18312687 PMC2275252

[B43] Quan-Xin F. Fan F. Xiang-Ying F. Shu-Jun L. Shi-Qi W. Zhao-Xu L. (2012). Resolvin D1 reverses chronic pancreatitis-induced mechanical allodynia, phosphorylation of NMDA receptors and cytokines expression in the thoracic spinal dorsal horn. BMC Gastroenterol. 12:148 10.1186/1471-230x-12-148 23092159 PMC3531273

[B44] Ramji N. Toth C. Kennedy J. Zochodne D. W. (2007). Does diabetes mellitus target motor neurons? Neurobiol. Dis. 26, 301–311 10.1016/j.nbd.2006.11.016 17337195

[B45] Scholz J. Woolf C. J. (2007). The neuropathic pain triad: neurons, immune cells and glia. Nat. Neurosci. 10, 1361–1368 10.1038/nn1992 17965656

[B46] Shehab S. A. Spike R. C. Todd A. J. (2004). Do central terminals of intact myelinated primary afferents sprout into the superficial dorsal horn of rat spinal cord after injury to a neighboring peripheral nerve? J. Comp. Neurol. 474, 427–437 10.1002/cne.20147 15174085

[B47] Steinbacher B. C. Jr. Nadelhaft I. (1998). Increased levels of nerve growth factor in the urinary bladder and hypertrophy of dorsal root ganglion neurons in the diabetic rat. Brain Res. 782, 255–260 10.1016/s0006-8993(97)01287-0 9519271

[B48] Sun R. Q. Lawand N. B. Willis W. D. (2003). The role of calcitonin gene-related peptide (CGRP) in the generation and maintenance of mechanical allodynia and hyperalgesia in rats after intradermal injection of capsaicin. Pain 104, 201–208 10.1016/s0304-3959(03)00008-3 12855330

[B49] Tan A. M. Chakrabarty S. Kimura H. Martin J. H. (2012). Selective corticospinal tract injury in the rat induces primary afferent fiber sprouting in the spinal cord and hyperreflexia. J. Neurosci. 32, 12896–12908 10.1523/jneurosci.6451-11.2012 22973013 PMC3499628

[B50] Tesfaye S. Boulton A. J. Dyck P. J. Freeman R. Horowitz M. Kempler P. (2010). Diabetic neuropathies: update on definitions, diagnostic criteria, estimation of severity and treatments. Diabetes Care 33, 2285–2293 10.2337/dc10-1303 20876709 PMC2945176

[B51] IJzerman T. H. Schaper N. C. Melai T. Meijer K. Willems P. J. Savelberg H. H. (2012). Lower extremity muscle strength is reduced in people with type 2 diabetes, with and without polyneuropathy and is associated with impaired mobility and reduced quality of life. Diabetes Res. Clin. Pract. 95, 345–351 10.1016/j.diabres.2011.10.026 22104262

[B52] Todd A. J. (2010). Neuronal circuitry for pain processing in the dorsal horn. Nat. Rev. Neurosci. 11, 823–836 10.1038/nrn2947 21068766 PMC3277941

[B53] Tong Y. G. Wang H. F. Ju G. Grant G. Hokfelt T. Zhang X. (1999). Increased uptake and transport of cholera toxin B-subunit in dorsal root ganglion neurons after peripheral axotomy: possible implications for sensory sprouting. J. Comp. Neurol. 404, 143–158 10.1002/(sici)1096-9861(19990208)404:2<143::aid-cne1>3.3.co;2-r 9934990

[B54] Wang H. B. Zhao B. Zhong Y. Q. Li K. C. Li Z. Y. Wang Q. (2010). Coexpression of delta- and mu-opioid receptors in nociceptive sensory neurons. Proc. Natl. Acad. Sci. U S A 107, 13117–13122 10.1073/pnas.1008382107 20615975 PMC2919913

[B55] Woolf C. J. (1995). Somatic pain–pathogenesis and prevention. Br. J. Anaesth. 75, 169–176 10.1093/bja/75.2.169 7577250

[B56] Yasuda H. Terada M. Maeda K. Kogawa S. Sanada M. Haneda M. (2003). Diabetic neuropathy and nerve regeneration. Prog. Neurobiol. 69, 229–285 10.1016/S0301-0082(03)00034-0 12757748

[B57] Zguira M. S. Vincent S. Le Douairon Lahaye S. Malarde L. Tabka Z. Saiag B. (2013). Intense exercise training is not effective to restore the endothelial NO-dependent relaxation in STZ-diabetic rat aorta. Cardiovasc. Diabetol. 12:32 10.1186/1475-2840-12-32 23399712 PMC3599941

[B58] Zuo Z. F. Wang W. Niu L. Kou Z. Z. Zhu C. Zhao X. H. (2011). RU486 (mifepristone) ameliorates cognitive dysfunction and reverses the down-regulation of astrocytic N-myc downstream-regulated gene 2 in streptozotocin-induced type-1 diabetic rats. Neuroscience 190, 156–165 10.1016/j.neuroscience.2011.06.025 21712075

